# Proteomic Insights into the Adaptation of *Acidithiobacillus ferridurans* to Municipal Solid Waste Incineration Residues for Enhanced Bioleaching Efficiency

**DOI:** 10.1021/acs.jproteome.4c00527

**Published:** 2025-04-09

**Authors:** Jiri Kucera, Klemens Kremser, Pavel Bouchal, David Potesil, Tomas Vaculovic, Dalibor Vsiansky, Georg M. Guebitz, Martin Mandl

**Affiliations:** 1 Department of Biochemistry, Faculty of Science, Masaryk University, Brno 625 00, Czech Republic; 2 University of Natural Resources and Life Sciences Vienna BOKU, Department of Agrobiotechnology, IFA-Tulln, Institute of Environmental Biotechnology, Tulln and der Donau 3430, Austria; 3 Proteomics Core Facility, Central European Institute for Technology, Masaryk University, Brno 625 00, Czech Republic; 4 Department of Chemistry, Faculty of Science, Masaryk University, Brno 625 00, Czech Republic; 5 Department of Geological Sciences, Faculty of Science, Masaryk University, Brno 611 37, Czech Republic

**Keywords:** *Acidithiobacillus*, adaptation, bioleaching, diaPASEF proteomics, metal recovery, municipal solid waste incineration residues

## Abstract

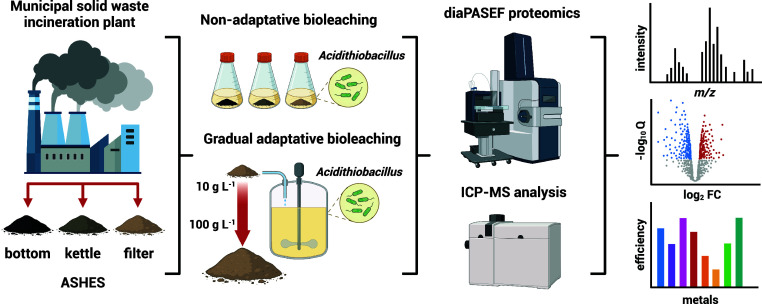

*Acidithiobacillus* spp. have traditionally been utilized to extract metals from mineral ores through bioleaching. This process has recently expanded to include artificial ores, such as those derived from municipal solid waste incineration (MSWI) residues. Previous studies have indicated that microbial adaptation enhances bioleaching efficiency, prompting this study to identify proteins involved in the adaptation of *A. ferridurans* to MSWI residues. We employed data-independent acquisition-parallel accumulation serial fragmentation to determine the proteomic response of *A. ferridurans* DSM 583 to three distinct materials: bottom ash (BA), kettle ash (KA), and filter ash (FA), which represent typical MSWI residues. Our findings indicate that, irrespective of the residue type, a suite of membrane transporters, porins, efflux pumps, and specific electron and cation transfer proteins was notably upregulated. The upregulation of certain proteins involved in anaerobic pathways suggested the development of a spontaneous microaerobic environment, which minimally impacted the bioleaching efficiency. Additionally, the adaptation was most efficient at half the target FA concentration, marked by a significant increase in the detoxification and efflux systems required by microorganisms to tolerate high heavy metal concentrations. Given that metal recovery peaked at lower FA concentrations for most metals of interest, further adaptation at the level of protein expression may not be warranted for improved bioleaching outcomes.

## Introduction

Municipal solid waste incineration (MSWI) residues and other industrial waste streams represent possible artificial ores for the urban mining of metals such as Cu, Ni, Zn, Mn, Al, Cd, and Cr.^1−3^ Incineration is one of the most commonly used waste treatment methods worldwide, and it produces a large amount of residual ash (up to 25% of the original waste volume), which is disposed without further recovery, thus resulting in the loss of the metals it contains. Although some attempts have been made to use MSWI residues in construction,^1,2^ their high metal concentration prevents direct use. Cost-effective methods of treating these residues to remove water-soluble salts and some metals include washing with water or acids. However, most MSWI residues are disposed into areas or underground landfills. Bioleaching using extremely acidophilic microorganisms can be a cost-effective and environmentally friendly method for extracting metals from these residues. Previous studies have reported the potential of bioleaching to recover metals from various industrial wastes, such as incineration residues,^3−5^ metallurgical slags and spent catalysts,^6−8^ and low-grade ores and deposits.^9,10^

Different strategies have been employed to enhance the efficiency of bioleaching of metals from artificial ores, mainly by optimizing the process parameters such as pulp density, pH, processing time, temperature, stirring speed, bioleaching method, and substrate availability. Furthermore, the adaptation of microorganisms to the selected waste material has likewise proven to be a key factor for effective bioleaching, with various studies reporting that microbial adaptation can enhance bioleaching of metals from waste materials such as incineration residues,^5^ metallurgical slags,^11^ or red mud.^12^ However, the underlying mechanism of such adaptation improving the efficiency of bioleaching of metals from waste materials is yet to be fully understood.

Mass spectrometry (MS)-based proteomics is a powerful tool for investigating changes in protein expression during microbial adaptation to different energy sources or stress conditions. *Acidithiobacillus ferrooxidans* is a model microorganism used in bioleaching processes, and several MS-based proteomic studies have evaluated its biofilm cell adaptation to pyrite,^13,14^ cellular response to high Cu^15−17^ and Cd^18^ concentrations, and cellular adaptation to an anaerobic environment.^19^ Recently, the adaptation of *A. ferrooxidans* to aerobic and anaerobic H_2_ metabolism^20^ and the response of *Acidihalobacter prosperus* to increased chloride concentration^21^ have been reported.

However, to the best of our knowledge, there have been no studies regarding the proteomic response of these microorganisms to artificial ore exposure. Therefore, this study aims to identify the proteins and pathways involved in the cellular response and adaptation of *A. ferridurans* to MSWI residues to explain the higher bioleaching efficiency of the adapted bacterial cells compared with that of the nonadapted bacterial cells.

## Materials and Methods

### MSWI Residues

MSWI residues such as bottom ash (BA, also referred to as slag), kettle ash (KA), and filter ash (FA) were obtained from a state-of-the-art MSWI plant in Austria that has a treatment capacity of ∼150,000 t residual waste and 50,000 t sewage sludge annually. Approximately 50 kg t^–1^ of fly ash is generated during incineration in this treatment plant, accounting for ∼10,000 t of fly ash annually. Raw BA, KA, and FA samples were washed and dried before use and characterized using inductively coupled plasma mass spectrometry (ICP-MS) and X-ray diffraction (XRD), as described previously.^5^ The pH value was measured according to ISO 10390.

### Chemical and Biological Leaching Conditions

*A. ferridurans* DSM 583 (formerly *A. ferrooxidans*(22)) was used for batch and continuous stirred tank reactor (CSTR) bioleaching experiments. The batch bioleaching of metals from different MSWI residues using nonadapted *A. ferridurans* cultures was performed in biological triplicates, as illustrated in Figure S1. First, 10 mL of fresh stationary culture (∼1 × 10^8^ cells mL^–1^) were precultivated in 250 mL shake flasks containing 90 mL of basal salt medium (BSM), comprising 0.4 g L^–1^ of (NH_4_)_2_SO_4_, 0.4 g L^–1^ of MgSO_4_ × 7 H_2_O, 0.4 g L^–1^ of KH_2_PO_4_, and 33.3 g L^–1^ of Fe_2_SO_4_ × 7 H_2_O, and adjusted to an initial pH of 1.4 using H_2_SO_4_. After 7 days, 10 mL of the grown culture was added to 90 mL of fresh Fe^2+^-containing BSM. Then, 10 g L^–1^ of heat-sterilized BA, KA, or FA and 10 g L^–1^ of heat-sterilized elemental sulfur (S^0^) were added to the flasks. Batch bioleaching was performed at 30 °C and 150 rpm on an orbital shaker. After 14 days, the cultures were centrifuged for 1 min at 1500 × *g* to remove S^0^ and the MSWI residues and stored at −80 °C for proteomic analyses.

Furthermore, the batch bioleaching of metals from FA using nonadapted and adapted *A. ferridurans* cultures was performed in biological triplicates, as illustrated in Figure S2. First, 10 mL of fresh stationary culture (∼1 × 10^8^ cells mL^–1^) was precultivated in 250 mL shake flasks containing 90 mL of Fe^2+^-containing BSM (initial pH = 1.4). After 7 days, 10 mL of the grown culture was added to 90 mL of fresh Fe^2+^-containing BSM. Then, 10 g L^–1^ of heat-sterilized FA and 10 g L^–1^ of heat-sterilized S^0^ were added to the flasks. Batch bioleaching was conducted at 30 °C and 150 rpm on an orbital shaker. After 14 days, 90 mL of the culture (designated as “nonadapted”) was filtered and stored at −80 °C for trace-element analysis. The remaining 10 mL of the culture was cultivated in 250 mL shake flasks containing 90 mL of fresh Fe^2+^-containing BSM supplemented with 10 g L^–1^ of heat-sterilized FA and 10 g L^–1^ of heat-sterilized S^0^. Batch bioleaching was conducted at 30 °C and 150 rpm on an orbital shaker. After 14 days, 100 mL of the culture (designated as “adapted”) was filtered and stored at −80 °C for trace-element analysis.

Following batch bioleaching, a scale-up to CSTR was performed, as illustrated in Figure S3. The reactor comprised a 2 L wide-mouth bottle with a working volume of 1.5 L connected to an M800 process control transmitter (Mettler Toledo). The pH was automatically adjusted to 2.4 using 2 M H_2_SO_4_ via the reactor transmitter connected to an acid pump and a pH electrode. Heating until 30 °C and continuous stirring at 250 rpm were performed using a heating plate (IKA) and an RCT basic magnetic stirring, respectively. Aeration of 60 L h^–1^ was performed using pressurized air. The reactor was operated semicontinuously, gradually increasing the FA concentration from 10 to 100 g L^–1^. Initially, the *A. ferridurans* culture was precultivated in the CSTR containing 1.5 L of Fe^2+^-containing BSM (initial pH = 1.4) to ensure sufficient biomass growth via Fe^2+^ oxidation. After 7 days, 10 g L^–1^ of heat-sterilized FA and 10 g L^–1^ of heat-sterilized S^0^ were added to the CSTR and the pH was adjusted to 2.4. After 14 days, 1 L (2/3) of the homogeneous culture was centrifuged for 1 min at 1500 × *g* to remove S^0^ and FA residues and stored at −80 °C for proteomic analyses. Optical density at 660 nm (OD_660_) of the sample was immediately determined before freezing. The sample was further filtered, and its pH was immediately determined, whereas the sample for trace-element analysis was stored at −80 °C. The remaining 0.5 L (1/3) of the culture was transferred to 1 L of fresh Fe^2+^-containing BSM supplemented with 10 g L^–1^ of heat-sterilized S^0^, to which FA was added to achieve a concentration of 25 g L^–1^. This procedure was further repeated to increase FA concentrations to 50, 75, and 100 g L^–1^, resulting in five adaptation cycles and a processing time of 11 weeks, as illustrated in Figure S3. At the end of each adaptation cycle, the samples were collected for proteomic and trace-element analyses and pH and OD_660_ measurements. Chemical leaching using only double-distilled water and H_2_SO_4_ to control pH was performed as the control abiotic leaching.

### Analysis of the Chemical and Biological Leaching Samples

Trace-element analysis was performed using ICP-MS, as described previously.^5^ The pH values were determined using a Mettler Toledo S220 pH meter with a combined glass electrode (Mettler Toledo). Before ICP-MS and pH measurements, all the samples were filtered using a 0.2 μm nylon CHROMAFIL Xtra filter (Macherey–Nagel) to remove cells, S^0^, and MSWI residues. Samples for cell density determination by OD_660_ measurement on a DR3900 spectrophotometer (Hach Lange) were not filtered but only briefly centrifuged for 1 min at 1500 × *g* to remove S^0^ and MSWI residues and preserve bacterial cells.

### Preparation of Biological Leaching Samples for Proteomic Analyses

Three biological replicates for each condition were performed for quantitative proteomic analysis. Protein extractions were performed as previously described.^20^ Briefly, 200 μL of lysis buffer containing 8 M urea in 100 mM M Tris–HCl (pH 7.5) was added to each bacterial pellet. The suspensions were homogenized through needle sonication (90 × 0.5 s pulses at 50 W; HD 2200, Bandelin) and then incubated for 60 min at 4 °C. The homogenates were centrifuged at 14,000 × *g* for 20 min at 4 °C. The aliquots of the supernatant containing 100 μg of total protein were processed using filter-aided sample preparation, as described elsewhere,^23^ using 1 μg of sequencing-grade trypsin (Promega). The resulting peptides were analyzed using liquid chromatography–tandem mass spectrometry (LC–MS/MS).

### LC–MS/MS Identification of Peptides

The LC–MS/MS analyses of all the peptides were performed using a nanoElute system (Bruker) connected to a timsTOF Pro spectrometer (Bruker). The single-column mode (separation column: Aurora C18, 75 μm ID, 250 mm long, 1.6 μm particles, IonOpticks) of the nanoElute system was used with default equilibration (separation column: four column volumes at 800 bar) and sample-loading (two pickup volumes + 2 μL at 800 bar) conditions. The concentrated peptides were eluted using an 80 min-long linear gradient program (flow rate = 300 nL min^–1^, 3–80% of mobile phase B; mobile phase A: 0.1% formic acid in water; mobile phase B: 0.1% formic acid in acetonitrile). The analytical column was placed inside the Column Toaster (40 °C; Bruker), and its emitter side was installed into the CaptiveSpray ion source (Bruker). MS data were acquired in the data-independent acquisition (DIA) mode with a base method *m*/*z* range of 100–1700 and a 1/k0 range of 0.6–1.6 V s cm^–2^. Table S1 defines the *m*/*z* 400–1000 precursor range with an equal window size of 30 Th using two steps for each parallel accumulation serial fragmentation (PASEF) scan and a cycle time of 100 ms locked to a 100% duty cycle.

### Processing of LC–MS/MS Data

DIA data were analyzed using Spectronaut software version 18.0 (Biognosys) in the direct DIA mode. The *A. ferridurans* DSM 583 genome (GCA_018854535.1), downloaded from NCBI on July 29, 2021, and containing 3435 protein-coding sequences, was used for the database search. Carbamidomethylation (C) was used as a fixed modification, and oxidation (M) and acetylation (protein N-term) were used as variable modifications. For protein identification, precursor and protein *Q*-value cutoffs (experiment level) were set to 0.01. Local data normalization was performed, and only the precursors identified in 3 of the 27 total runs (0.11 percentile precursor filtering) were included in the final data set. A global imputation strategy was used. Default settings were used for other parameters. Single-hit proteins were excluded. Proteins with |log_2_ fold change| of >1 and a *Q*-value of <0.05 (calculated using unpaired *t*-test as implemented in Spectronaut 18.0, including false discovery rate correction) were considered differentially expressed proteins (DEPs). The STRING database version 12.0^24^ was employed for the functional enrichment analysis using Gene Ontology. The volcano and upset plots were drawn using ChiPlot (http://www.chiplot.online). The MS proteomics data have been deposited to the ProteomeXchange Consortium via the PRIDE^25^ partner repository with the data set identifier PXD053126.

### Statistical Analyses

Statistical analyses were performed using Prism v10.2.2 (GraphPad Software). All experimental data are presented as mean ± SD and were analyzed using unpaired *t*-test.

## Results and Discussion

### Nonadaptive Batch Bioleaching

#### Proteomic Analysis Overview

Using DIA–PASEF (diaPASEF) analysis of *A. ferridurans* DSM 583 cells, 2017 proteins were quantified (coverage 58.7%; a total of 3435 protein-coding sequences) based on at least two proteotypic peptides in 11% measurements (3 of 27) across the study (Table S2). This new approach identified a higher number of proteins than our previous DIA–MS analysis of *A. ferrooxidans* CCM 4253, which quantified 1412 proteins (coverage 46.2%; a total of 3059 protein-coding genes) based on a single proteotypic peptide.^20^

**Figure 1:**
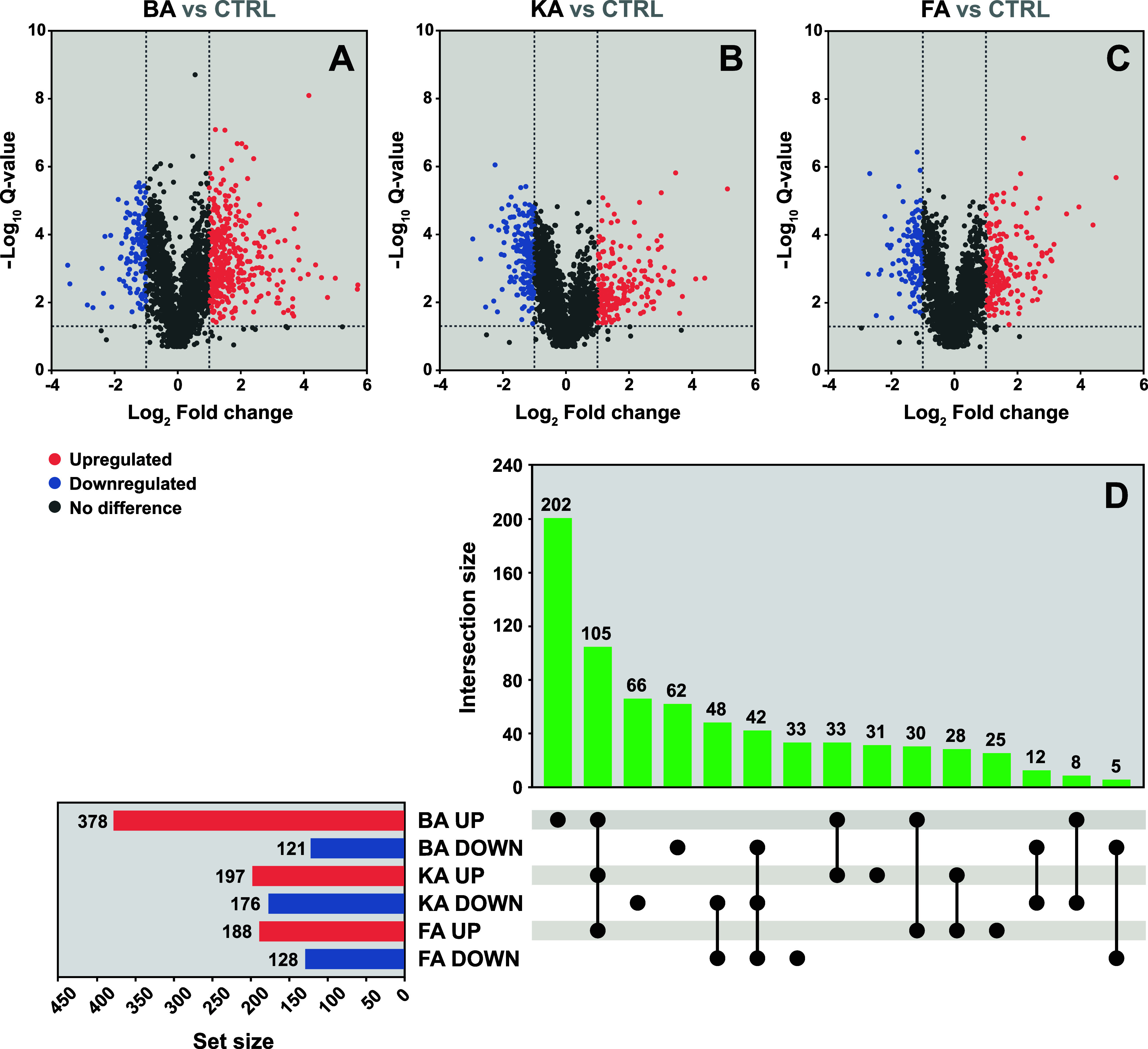
Proteomic profiles of nonadapted *A. ferridurans* cultures during batch bioleaching of metals from various types of municipal solid waste incineration (MSWI) residues. A detailed experimental design is shown in Figure S1. Table S3 contains a list of differentially expressed proteins. Volcano plots representing all the identified proteins in the cultures containing (A) bottom ash (BA), (B) kettle ash (KA), and (C) filter ash (FA) compared with those in the control cultures without MSWI residues (CTRL). Colored circles indicate differentially expressed proteins (DEPs) that changed significantly (|log_2_ fold change| > 1 and *Q*-value <0.05); upregulated and downregulated DEPs are colored red and blue, respectively. The upset plot compares the number of DEPs and their relationship (D).

The proteomes of the nonadapted *A. ferridurans* cultures after the batch bioleaching of metals from various MSWI residues were compared with those of the control *A. ferridurans* cultures grown without MSWI residues. The MSWI residues have been previously characterized, and the bioleaching efficiency of *A. ferridurans* has been reported.^5^ The DEPs in the presence of various MSWI residues are listed in Table S3. A comparison with the control *A. ferridurans* cultures revealed that in the presence of 10 g L^–1^ of BA, 378 and 121 proteins were upregulated and downregulated, respectively (Figure 1A); in the presence of 10 g L^–1^ of KA, 197 and 176 proteins were upregulated and downregulated, respectively (Figure 1B); and in the presence of 10 g L^–1^ of FA, 188 and 128 proteins were upregulated and downregulated, respectively (Figure 1C). In the presence of all the MSWI residues, 105 and 42 shared proteins were upregulated and downregulated (Figure 1D), respectively. Furthermore, 202 proteins were exclusively upregulated in the presence of BA, 31 in the presence of KA, and 25 in the presence of FA (Figure 1D). Conversely, 62 proteins were exclusively downregulated in the presence of BA, 66 in the presence of KA, and 33 in the presence of FA (Figure 1D). On comparing the protein profiles of *A. ferridurans* exposed to different MSWI residues, those of *A. ferridurans* exposed to FA and KA were more related to each other than those of *A. ferridurans* exposed to BA, which was more divergent (Figure S4). This could be attributed to the composition of these artificial ores. BA contained twice as much Fe, Co, and Ni as KA and FA, while the Zn and Cd contents were considerably higher in KA and FA than those in BA.^5^

#### Functional Enrichment in Biological Processes

**Figure 2:**
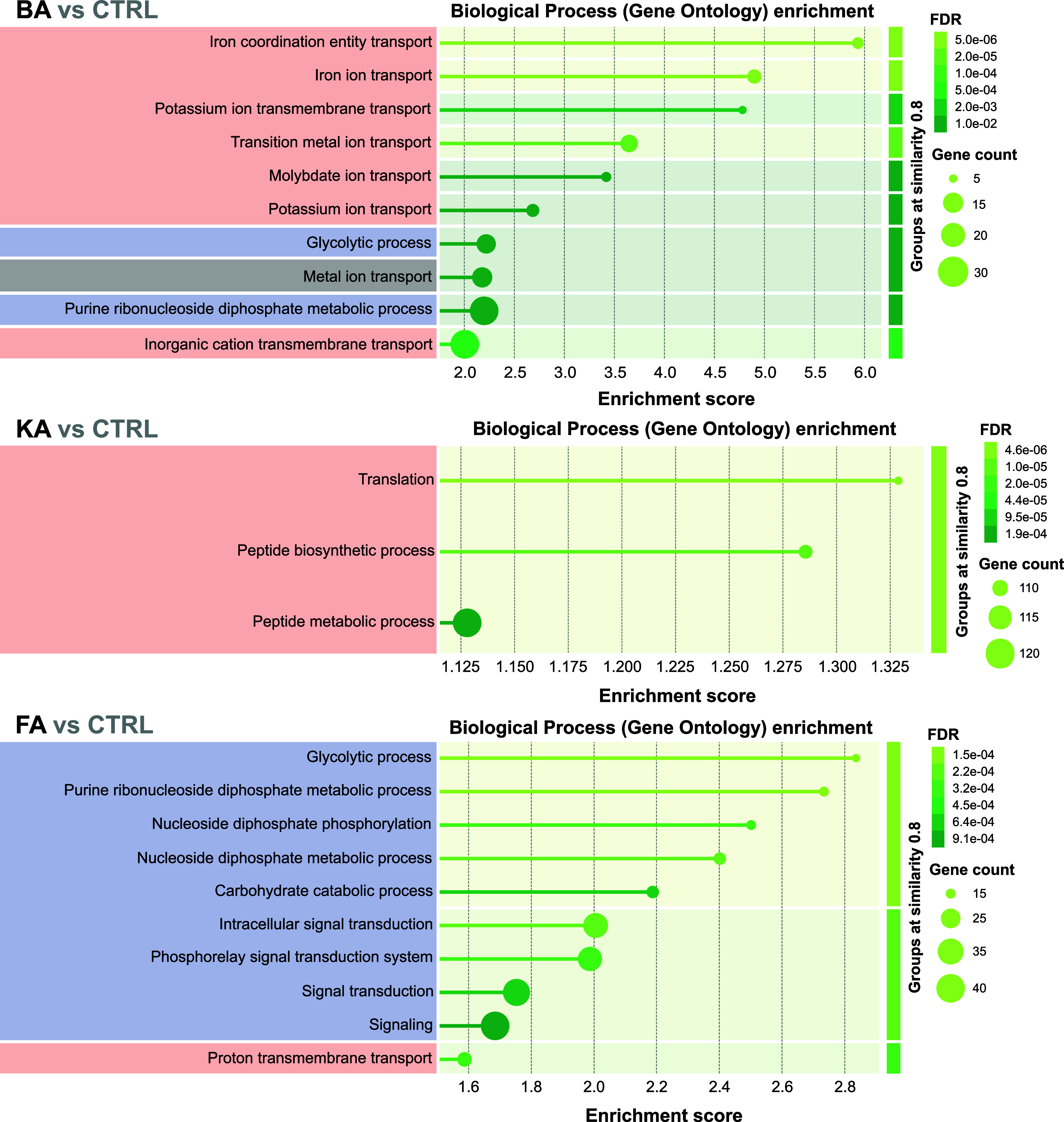
Gene Ontology (GO) enrichment analysis of nonadapted *A. ferridurans* cultures during batch bioleaching of metals from bottom ash (BA), kettle ash (KA), and filter ash (FA). The detailed outcomes of the functional enrichment analysis are shown in Table S4. The color-coded GO terms represent upregulation (red), downregulation (blue), and no differences (gray).

Nonadaptive batch bioleaching resulted in the upregulation of proteins involved in biological processes, including the transport of inorganic cations (potassium, iron, and other transition metals) and anions (molybdate) in BA, translation and peptide biosynthesis in KA, and proton transport in FA (Figure 2). It appears that increased potassium transport helps *A. ferridurans* maintain pH stability by counteracting proton influx, stabilizing osmotic pressure, and activating transport systems, such as efflux pumps that export toxic metals from the cell. Additionally, potassium helps stabilize enzyme function, enabling the bacteria to sustain metabolic activities crucial for bioleaching processes, such as sulfur and iron oxidation. Molybdate is an essential compound in bacteria, primarily providing molybdenum, a critical cofactor for various enzymes involved in redox reactions, including those in respiration, electron transfer, and sulfur and nitrogen metabolism. The elevated production of peptides and proteins in the presence of KA likely reflects the activation of adaptive pathways that enable *A. ferridurans* to survive and thrive in harsh bioleaching environments characterized by high acidity and heavy metal concentrations. On the other hand, downregulating energy-intensive pathways, such as glycolysis, nucleotide metabolism, and signal transduction, helps *A. ferridurans* conserve energy and redirect it to essential survival functions like membrane integrity, metal detoxification, and pH homeostasis. This downregulation limits DNA damage under metal stress and reduces proton influx, supporting survival in acidic, metal-rich bioleaching environments. Overall, these adjustments reflect an adaptive shift from growth-focused processes to stress resistance and inorganic energy metabolism.

#### In-Depth Analysis of Proteins and Their Functional Roles

During batch bioleaching, the nonadapted *A. ferridurans* cultures oxidized Fe^2+^ irrespective of the type of the MSWI residue, as demonstrated by the upregulation of proteins involved in electron transfer in the aerobic Fe^2+^ oxidation pathway. The redox proteins encoded by the *rus* (*c*-type cytochromes Cyc2 and Cyc1, cupredoxins Rus and AcoP, and cytochrome *c* oxidase CoxBACD) and *petI* (cytochrome *bc*_1_ complex PetA1B1C1 and *c*-type cytochrome CycA1) operons, which are responsible for electron transfer from Fe^2+^ to O_2_ or NAD^+^, have been exclusively detected in *Acidithiobacillus* spp.^22,26−30^ Our results supported those of a previously published report, wherein genetically engineered AcoP-overproducing *A. ferrooxidans* cells exhibited better Cu recovery from BA compared to the wild-type strain.^31^ Interestingly, the redox proteins encoded by *rus*-like (Cyc1- and Cyc2-like) and *petII* (PetA2B2C2, CycA2, and high potential Fe–S protein Hip) operons, which are involved in the anaerobic Fe^3+^ reduction pathway in *Acidithiobacillus* spp.,^19,20,32−34^ were upregulated. The upregulation of the proteins involved in the anaerobic respiratory pathway suggests that a spontaneous microaerobic environment had evolved owing to the increased O_2_ demands of the growing biomass that were not adequately met by agitation and air supply. In addition, it is consistent with the observed upregulation of cytochrome *bd* ubiquinol oxidase. The levels of this enzyme increase when O_2_ becomes limiting, as it has a higher affinity for O_2_ than cytochrome *c* oxidase encoded by the *rus* operon.^35^ Under batch bioleaching conditions, Fe recycling occurred in the spontaneous microaerobic environment, thereby upregulating the proteins involved in the Fe^2+^ oxidation and Fe^3+^ reduction pathways compared with those in the control cultures, which oxidized only Fe^2+^ in the absence of MSWI residues. In addition, Fe was released from the MSWI residues, further upregulating the redox proteins involved in these pathways. Although the oxidation of S^0^ to H_2_SO_4_ occurred during batch bioleaching, as indicated by a drop in pH, several proteins involved in reduced inorganic sulfur compound (RISC) oxidation were downregulated; these proteins belong to the *hdr* operon encoding heterodisulfide reductase and additional sulfur transporters such as rhodanese, TusA, and DsrE proteins.^36^

Additionally, the cell envelope structure of the nonadapted *A. ferridurans* cultures changed significantly. Porin-like major outer membrane proteins (OmpA and OmpH), which play a structural role in bacterial cell surface integrity,^37^ and several outer membrane channels (TolC), which span the outer membrane and periplasm of Gram-negative bacteria, were upregulated, enabling the transport of small molecules and heavy metals.^38^ CusC is one of three TolC orthologues in *Escherichia coli* that is involved in the efflux of toxic Cu^+^ and Ag^+^ ions.^39^ An important role of TolC family proteins in heavy metal resistance was demonstrated by the deletion of the *tolC* gene in *Riemerella anatipestifer*, inducing hypersensitivity to metals such as Mn^2+^, Zn^2+^, Cu^2+^, Fe^2+^, Co^2+^, and Ni^2+^.^40^ TonB-dependent receptors, which are associated with the uptake and transport of large substrates such as Fe–siderophore complexes in Gram-negative bacteria,^37^ were also upregulated. Furthermore, there were changes in the expression of cytoplasmic membrane transporters such as resistance–nodulation–division (RND) family efflux transporters, ATP-binding cassette (ABC) superfamily, and major facilitator superfamily (MFS), which can transport various toxic substances as a cellular self-defense mechanism.^41,42^ The RND-driven efflux complexes reportedly play an essential role in the Cu resistance of *Acidithiobacillus* spp.^43^ RND proteins (CusBA) cooperate with other proteins belonging to a family of outer membrane factors, such as the TolC protein family (CusC), forming an efflux protein complex CusCBA that can transport its substrate from the cytoplasm, cytoplasmic membrane, or the periplasm across the outer membrane directly outside the cell. The action of RND proteins is driven by protons that were previously pumped out by the respiratory chain.^44^ Most bacterial ABC transporters comprise transmembrane permeases, nucleotide-binding proteins, and specific periplasmic solute-binding proteins. We detected an upregulation in the phosphate ABC transporter substrate-binding protein PstS, which belong to the *pst* operon that encodes the protein-dependent Pst transport system for inorganic phosphate uptake in numerous bacterial species.^45^ Polyphosphates can also sequester heavy metals in *Acidithiobacillus* spp. Some metal cations stimulate the activity of polyphosphatase, leading to the release of phosphate from polyphosphates, following which MeHPO_4_^–^ ions are transported out of the cells.^43^ Previous reports on metal resistance in *Sulfolobus metallicus* have suggested the presence of phosphate–proton symporters such as Pho84-like protein belonging to the MFS superfamily.^43^ Thus, a higher phosphate uptake possibly contributed to the enhanced tolerance of *A. ferridurans* to heavy metals during the batch bioleaching of metals from MSWI residues.

Moreover, heavy metal cations have different affinity levels for thiol compounds, such as reduced glutathione, which is related to the toxicity of each heavy metal cation.^44^ However, the situation is more complex in *Acidithiobacillus* spp., as intermediates of RISC metabolism can form metal sulfides. Gram-negative bacteria contain an intracellular glutathione pool, and metals that are not required in the active centers of enzymes are present in glutathionate complexes,^44^ which may explain the upregulation of glutathione-*S*-transferase. The proteins involved in the fate of glutathione were reportedly upregulated in the presence of Cu.^15^ The resulting redox imbalance likely caused changes in the levels of thioredoxins, and proteins involved in the fate of glutathione.^46−48^ Additionally, protein disulfide reductase DsbD and thiol:disulfide interchange protein DsbG were upregulated, which can help restore native disulfide bridges, thereby allowing the survival of acidophiles under heavy metal stress.^15^ The cysteine residues in protein-binding sites and high energy are required to detoxify heavy metal cations bound to thiols.^44^

A few P-type ATPases, which meet the above requirements and whose substrates are metal–thiolate complexes in *E. coli*,^49^ were significantly upregulated during nonadaptive bioleaching. In *A. ferrooxidans*, Cu-translocating P-type ATPases (CopA and CopB) act as resistance factors for Cu ions.^50^ The nonadapted cultures responded to the elevated concentration of metals released from the MSWI residues during batch bioleaching by regulating multiple layers of metal resistance to enhance effective metal sensing and efflux.

#### Impact of Adaptation on Batch Bioleaching

The efficiency of batch bioleaching using nonadapted and adapted *A. ferridurans* cultures supplemented with 10 g L^–1^ of FA was compared. XRD mineral phase analysis revealed that the metals present in FA were primarily in the form of oxides or sulfates (Table 1), consistent with the findings of previous studies on waste incineration residues.^5,51^ FA exhibited high alkalinity, with a pH of 10.13 and a dry matter content of 96.7%. The concentrations of the selected metals in untreated FA were as follows: 45.20 g kg^–1^ of Al, 0.09 g kg^–1^ of Cd, 0.04 g kg^–1^ of Co, 0.22 g kg^–1^ of Cr, 6.04 g kg^–1^ of Cu, 0.91 g kg^–1^ of Mn, 0.11 g kg^–1^ of Ni, 0.07 g kg^–1^ of V, 7.42 g kg^–1^ of Zn, and 20.60 g kg^–1^ of Fe. ICP-MS analysis revealed economically attractive Al, Zn, Cu, Mn, and Cr concentrations, ranging from 0.22 to 45.2 g kg^–1^. For Cu, the concentration of 6.04 g kg^–1^ in the MSWI residue exceeded that in the ores used for commercial mineral extraction, in which the ore grade declines.^52,53^

**Table 1:** Phase Composition of Untreated Filter Ash

mineral	chemical formula	wt %
amorphous		45.4
anhydrite	CaSO_4_	9.1
alkali feldspar	(Na,K)AlSi_3_O_8_	3.5
basanite	2CaSO_4_·H_2_O	3.0
calcite	CaCO_3_	2.4
cristobalite	SiO_3_	0.1
eckermannite	Ca_2_MgSi_2_O_7_	9.3
halite	NaCl	6.4
hematite	α-Fe_2_O_3_	1.0
hydroxylellestadite	Ca_5_(SiO_4_,SO_4_)_3_(OH,Cl,F)	6.0
larnite	β-Ca_2_SiO_4_	4.3
maghemite	γ-Fe_2_O_3_	1.2
magnetite	Fe_3_O_4_	0.2
sylvite	KCl	3.0
quartz	SiO_2_	2.3

**Figure 3:**
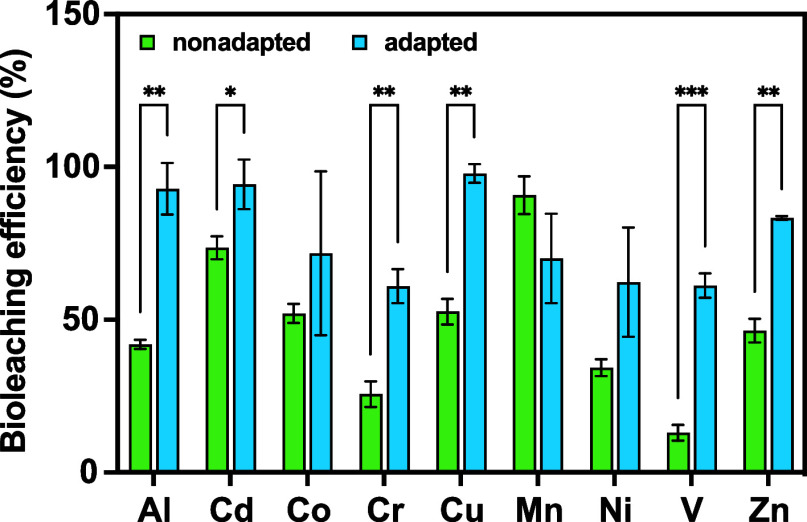
Bioleaching efficiency in the batch experiments. A detailed experimental design is shown in Figure S2. The nonadapted (green) and adapted (blue) *A. ferridurans* cultures supplemented with 10 g L^–1^ of filter ash (FA) were compared. The adapted culture was precultured in the presence of 10 g L^–1^ of FA. Significant differences were assessed via unpaired *t*-test and are indicated by *P_adj_ < 0.05, **P_adj_ < 0.01, and ***P_adj_ < 0.001. Error bars indicate the standard deviation (SD, *n* = 3).

**Figure 4:**
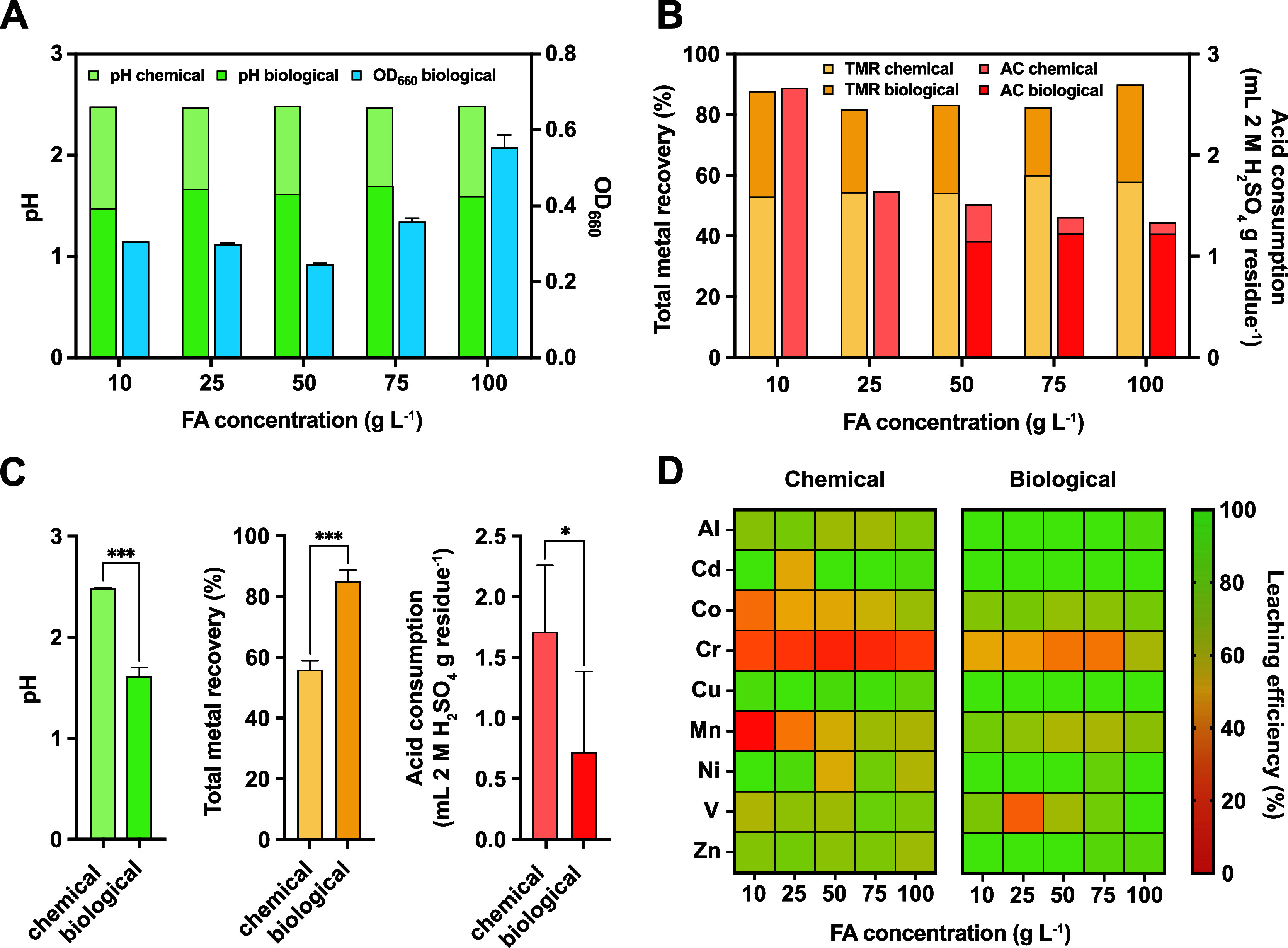
Chemical and biological leaching in a semicontinuous stirred tank reactor using gradually adapted *A. ferridurans* cultures. A detailed experimental design is shown in Figure S3. (A) pH and OD_660_ at the end of each adaptation cycle for chemical (light green) and biological (green and blue, respectively) leaching. (B) Total metal recovery (TMR) and acid consumption (AC) at the end of each adaptation cycle for chemical (light yellow and light red, respectively) and biological (yellow and red, respectively) leaching. (C) Overall comparison of chemical and biological leaching during all the adaptation cycles in terms of pH (green), TMR (yellow), and AC (red). (D) Heat maps showing chemical (left) and biological (right) leaching efficiency for the monitored metals during the adaptation cycles in response to increasing filter ash concentrations. Significant differences were assessed via unpaired *t*-test and are indicated by *P_adj_ < 0.05 and ***P_adj_ < 0.001. Error bars indicate the standard deviation (SD, *n* = 3).

The adapted culture was first precultured in the presence of the same amount of 10 g L^–1^ of FA. After 14 days, a difference was observed in the bioleaching efficiency of the adapted cultures for the tested metals (Figure 3). The adapted cultures showed significantly enhanced bioleaching efficiency for six metals (*P*_adj_ < 0.05) compared with nonadapted culture. The bioleaching efficiency of the adapted cultures for V increased almost fivefold, and that for Al and Cr increased more than twofold compared to nonadapted cultures. The adapted cultures exhibited >60% bioleaching efficiency for all the metals, with that for Al, Cd, Cu, and Zn reaching >80% (Figure 4).

### Adaptive Semicontinuous Bioleaching in the CSTR

#### Bioleaching Efficiency and Metal Recovery

Since the adaptation had a positive impact, semicontinuous bioleaching in the CSTR was performed following the batch bioleaching phase, wherein the FA concentration was gradually increased to enable the *A. ferridurans* cells to adapt to higher FA concentrations. Independent of FA concentration, microbial growth decreased the pH value to ∼1.5 due to S^0^ oxidation within 2 weeks of each adaptation cycle, whereas the abiotic control consistently maintained a pH of ∼2.4 (Figure 4A). Moreover, the OD_660_ values revealed that the bacterial cell density gradually increased from that at 50 g L^–1^ of FA to higher concentrations (Figure 4A). This resulted in lower acid consumption during bioleaching, as no additional H_2_SO_4_ was required to adjust the pH at initial concentrations of 10–25 g L^–1^ of FA (Figure 4B). In addition, the amount of H_2_SO_4_ required was also less when using FA concentrations of 50–100 g L^–1^, resulting in more than twofold lower total acid consumption in biological leaching compared with that in chemical leaching. The total metal recovery during leaching in the CSTR did not significantly alter during the adaptation cycles, ranging 80–90% for biological leaching and 50–60% for chemical leaching (Figure 4B). The difference in the total metal recovery and final pH between biological and chemical leaching during the five adaptation cycles was highly significant (*P*_adj_ < 0.001), along with the difference in the total acid consumption (*P*_adj_ < 0.05; Figure 4C). Although the total metal recovery did not differ significantly during gradual adaptation, certain metals, such as Al, Cd, Cu, Ni, and Zn, were biologically extracted at a rate of 100% in almost all the adaptation cycles of bioleaching in the CSTR regardless of FA concentration (Figure 4D). Interestingly, 100% bioleaching efficiency for Al, Cd, Cu, Ni, and Zn was achieved after the first week during the adaptation cycle in the presence of 10 g L^–1^ of FA, potentially reducing the process time. Thus, gradual adaptation influenced the increase of bioleaching efficiency for the selected metals Co, Cr, and especially V (Figure 4D).

#### Proteomic Analysis Overview

**Figure 5:**
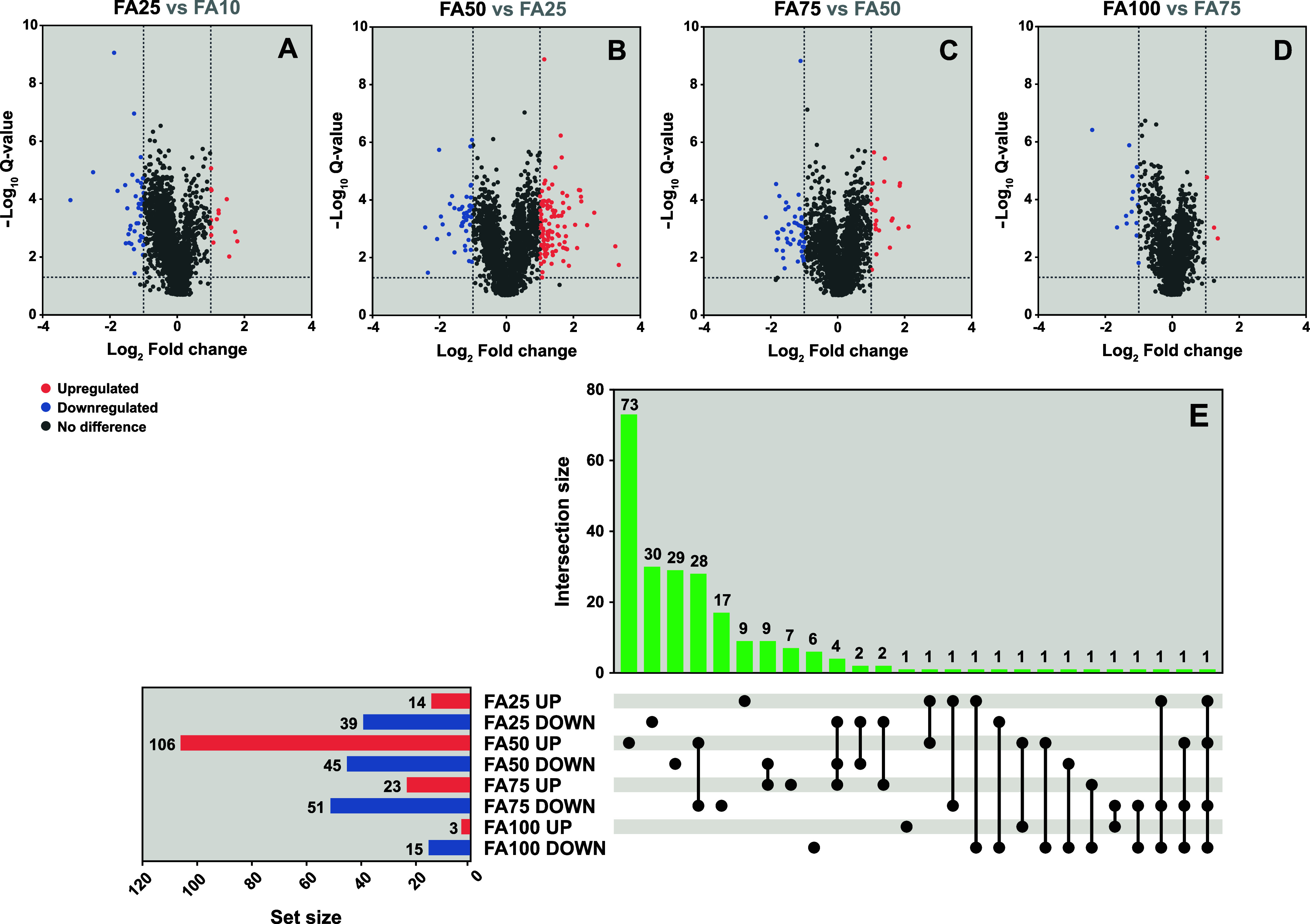
Proteomic profiles of gradually adapted *A. ferridurans* cultures during semicontinuous bioleaching of metals from increasing filter ash (FA) concentrations. A detailed experimental design is shown in Figure S3. Table S3 contains a list of differentially expressed proteins. Volcano plots representing the identified proteins in the cultures in the presence of (A) 25 g L^–1^ of FA compared with those in the cultures in the presence of 10 g L^–1^ of FA, (B) 50 g L^–1^ of FA compared with those in the cultures in the presence of 25 g L^–1^ of FA, (C) 75 g L^–1^ of FA compared with those in the cultures in the presence of 50 g L^–1^ of FA, and (D) 100 g L^–1^ of FA compared with those in the cultures in the presence of 75 g L^–1^ of FA. Colored circles indicate differentially expressed proteins (DEPs) that changed significantly (|log_2_ fold change| > 1 and *Q*-value <0.05); upregulated DEPs are colored red, and downregulated DEPs are colored blue. The upset plot compares the number of DEPs and their relationship (E).

The protein profiles of the gradually adapted *A. ferridurans* cultures were compared during the semicontinuous bioleaching of metals from different concentrations of FA from low to high. The DEPs in response to increasing FA concentrations are listed in Table S3. Total of 14 and 39 proteins were upregulated and downregulated, respectively, in the presence of 25 g L^–1^ of FA compared with that in the presence of 10 g L^–1^ FA (Figure 5A). Furthermore, 106 and 45 proteins were upregulated and downregulated, respectively, in the presence of 50 g L^–1^ of FA compared with that in the presence of 25 g L^–1^ of FA (Figure 5B); 23 and 51 proteins were upregulated and downregulated, respectively, in the presence of 75 g L^–1^ of FA compared with that in the presence of 50 g L^–1^ of FA (Figure 5C). Only three proteins were upregulated, and 15 were downregulated in the presence of 100 g L^–1^ of FA compared with that in the presence of 75 g L^–1^ of FA (Figure 5D). Nine proteins were exclusively upregulated in the presence of 25 g L^–1^ of FA, 73 proteins were exclusively upregulated in the presence of 50 g L^–1^ of FA, seven proteins were exclusively upregulated in the presence of 75 g L^–1^ of FA, and only one protein was exclusively upregulated in the presence of 100 g L^–1^ FA (Figure 5E). In addition, 30 proteins were exclusively downregulated in the presence of 25 g L^–1^ of FA, 29 proteins were exclusively downregulated in the presence of 50 g L^–1^ of FA, 17 proteins were exclusively downregulated in the presence of 75 g L^–1^ of FA, and 6 proteins were exclusively downregulated in the presence of 100 g L^–1^ of FA (Figure 5E).

When comparing the protein profiles of *A. ferridurans* exposed to increasing concentrations of FA, it is evident that the higher concentrations of 75–100 g L^–1^ are more similar than lower 10–25 g L^–1^ (Figure S4). This is likely due to the primary adaptation occurring at a concentration of 50 g L^–1^ and significant differences in the heavy metal content.

#### Functional Enrichment in Biological Processes

**Figure 6:**
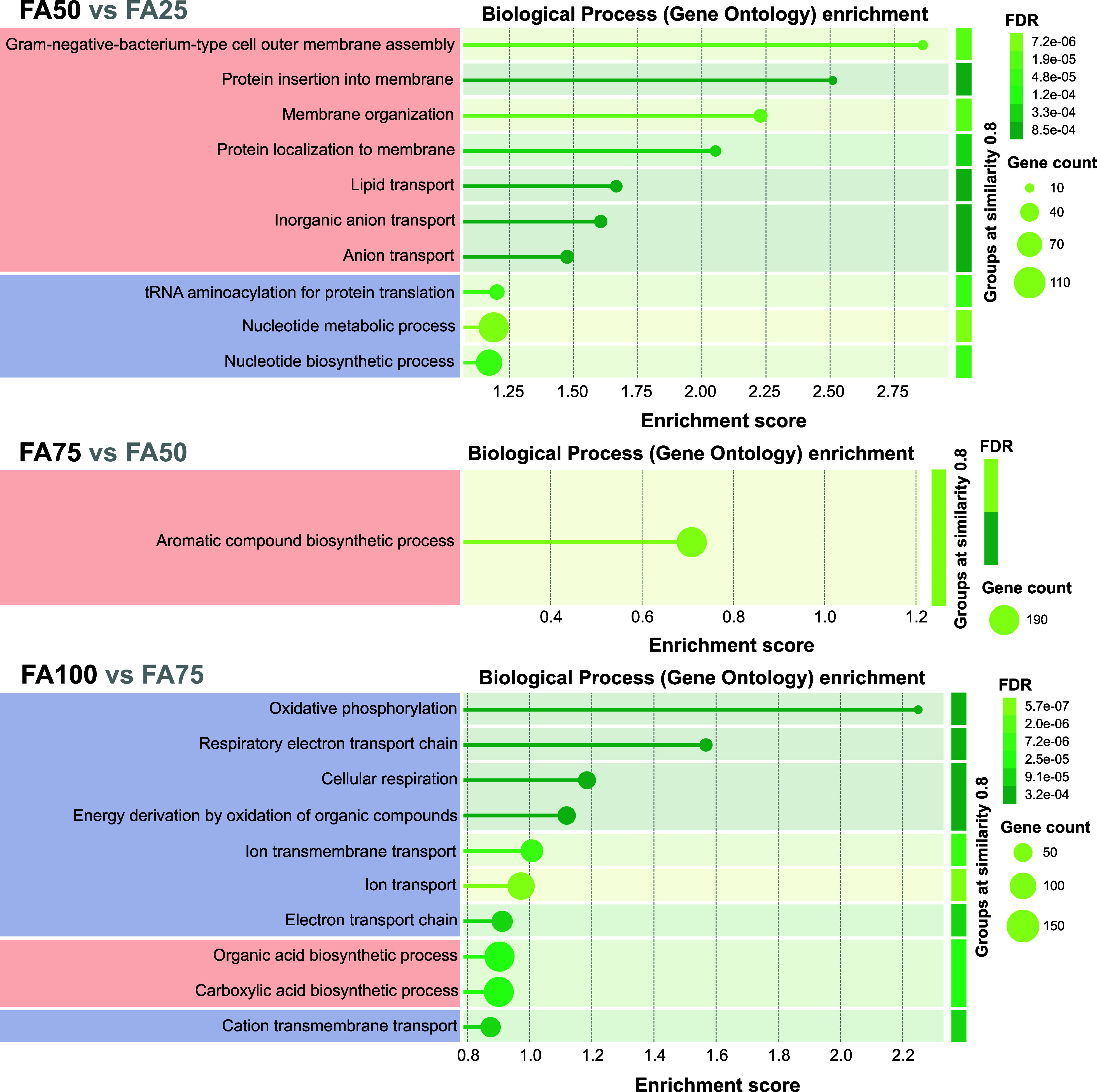
Gene Ontology (GO) enrichment analysis of gradually adapted *A. ferridurans* cultures during semicontinuous bioleaching of metals from increasing filter ash (FA) concentrations. The detailed outcomes of the functional enrichment analysis is shown in Table S4. The color-coded GO terms represent upregulation (red) and downregulation (blue).

Adaptive semicontinuous bioleaching resulted in the upregulation of proteins involved in biological processes, including outer membrane assembly and organization, protein insertion and localization to membrane, and transport of lipid and inorganic anions in the presence of 50 g L^–1^ of FA, aromatic compound biosynthesis in the presence of 75 g L^–1^ of FA, and organic acid biosynthesis, such as carboxylic acids, in the presence of 75 g L^–1^ of FA (Figure 6). By producing more organic acids, *A. ferridurans* can sequester toxic metals like Fe, Cu, and Zn, reducing their reactivity and protecting cellular structures from metal-induced damage. Additionally, organic acids may be involved in synthesizing key coenzymes or metabolic intermediates required for iron and sulfur metabolism, supporting energy production from inorganic compounds, and activate cellular defenses, boosting resilience in metal-rich, acidic environments. On the other hand, downregulating energy-intensive processes like protein translation and nucleotide biosynthesis helps *A. ferridurans* conserve energy, allowing it to focus on key survival functions such as metal detoxification and inorganic energy acquisition under resource-limited conditions. This resource allocation prioritizes stress response proteins, such as metal transporters and detoxification enzymes, while reducing DNA replication to minimize errors and maintain cellular integrity. Overall, downregulation reflects a shift toward survival rather than growth.

#### In-Depth Analysis of Proteins and Their Functional Roles

Cell adaptation is essential for enhancing the bioleaching efficiency of natural and artificial ores.^5,54−57^ We confirmed that the adapted *A. ferridurans* cultures exhibited higher bioleaching efficiency in FA than the nonadapted ones for most of the selected metals (Figure 3). To further characterize the adaptive processes, changes in the protein profiles of *A. ferridurans* cultures during gradual adaptation to increasing FA concentrations were investigated (Figures 4–6).

During the second adaptive bioleaching cycle at a concentration of 25 g L^–1^ of FA, the proteins involved in lipopolysaccharide synthesis were upregulated, likely leading to the formation of new extracellular polymeric substances. Cyclic beta-1,2-glucan synthetase is an enzyme responsible for the synthesis of cyclic beta-1,2-glucans, which are important polysaccharides involved in bacterial signaling, osmotic regulation, and biofilm formation^58^ and are thus vital for bacterial survival and stress response. Additionally, MFS and ABC transporters, TonB-dependent receptor, and metal cation transporters were upregulated.

The most extensive proteomic changes occurred during adaptive bioleaching at half the target FA concentration. The RND, ABC, TolC, and TonB family proteins were again significantly upregulated during the third adaptive bioleaching cycle at an FA concentration of 50 g L^–1^. In addition, a further upregulation of the proteins involved in lipopolysaccharide metabolism and transport, as well as novel transporters and porins of unknown function, were observed. Although increased agitation and ample air supply were ensured compared with those during batch bioleaching, a microaerobic environment could evolve again at this FA concentration, manifesting as the upregulation of several redox proteins involved in the anaerobic Fe^3+^ reduction pathway, as described above. Interestingly, formate hydrogenlyase was among the upregulated proteins. This enzyme is predicted to be associated with the group 4 hydrogenase (Hyf) complex, which forms a formate hydrogenase supercomplex in *A. ferrooxidans*.^59^ This supercomplex catalyzes the disproportionation of formate to CO_2_ and H_2_. This formate-dependent H_2_ production during adaptive bioleaching may explain the spontaneous evolution of a microaerobic environment, resulting in the upregulation of respiratory and uptake Ni-dependent hydrogenases, which typically oxidize H_2_ and transport the resulting electrons to quinones or cytochromes, respectively.^20,59^

Putative sulfide-quinone reductase and heterodisulfide reductase subunit HdrA were upregulated during the fourth adaptive bioleaching cycle at a concentration of 75 g L^–1^ of FA, suggesting increased RISC oxidation at the late stage of adaptation. Moreover, some proteins that induce structural changes in DNA were upregulated. Bacterial adaptation to multiple stresses caused by such high FA concentrations, implying high levels of heavy metal ions, may have led to random DNA mutations that needed to be localized and repaired to maintain genome integrity and ensure cell survival. Additionally, formate hydrogenlyase and cytochromes involved in anaerobic Fe^3+^ reduction were downregulated, suggesting that neither H_2_ oxidation nor Fe^3+^ reduction occurred any longer and that O_2_ did not limit bioleaching.

The last adaptive bioleaching cycle at an FA concentration of 100 g L^–1^ revealed only minimal changes at the protein level. All three proteins are poorly characterized, containing domains such as beta-lactamase domain and tetratricopeptide repeat sequence. Putative molybdopterin synthase was upregulated, which may have led to an increase in molybdopterin production, contributing to heavy metal removal in the cell.^60^ Besides the divalent metal cation and MFS transporters, the NADH oxidoreductase NuoM was downregulated, which may reduce the generation of reducing equivalents for CO_2_ fixation, resulting in limited growth. Additionally, PstA was significantly downregulated, indicating a reduction in phosphate uptake.

## Conclusions

Herein, the proteomic response of extremely acidophilic *A. ferridurans* to various MSWI residues during batch bioleaching was characterized. Different outer and inner membrane transporters were involved in the resistance mechanisms of *A. ferridurans* to heavy metals. In addition, an upregulation of the redox proteins involved in the aerobic oxidation of Fe^2+^ to Fe^3+^ was observed, allowing oxidative dissolution together with H_2_SO_4_ generation via S^0^ oxidation. However, an upregulation of the redox proteins involved in the anaerobic reduction of Fe^3+^ to Fe^2+^ was also observed, indicating the evolution of a spontaneous microaerobic environment that enables reductive dissolution, which is favorable, albeit operationally more challenging, for metal oxides such as those present in MSWI residues. In addition, microaerobic conditions can reduce oxidative stress, which has a detrimental effect on the cell. The maximum effect of gradual adaptation occurred as early as at half the target concentration of FA (at 50 of 100 g L^–1^ of FA). At a concentration of 50 g L^–1^ of FA, the stepwise upregulation of efflux transporters, which are required for adaptation to high metal concentrations, was the highest. Moreover, metal recovery was the highest for most selected metals at FA concentrations of 10–25 g L^–1^. Further adaptation to 75–100 g L^–1^ of FA appeared unnecessary in terms of protein expression and metal recovery. At such high concentrations, less significant changes occurred at the protein level of *A. ferridurans*; however, undesirable changes may have occurred at the DNA level. As there was no significant change in the total metal recovery with gradual adaptation, but there was a significant change in the recovery of individual metals, this is mostly related to different layers of heavy metal resistance of *A. ferridurans*.

## Data Availability

Data are available via ProteomeXchange with identifier PXD053126.

## Supporting Information Available

(Figure S1) Batch bioleaching of metals from MSWI residues using nonadapted *A. ferridurans* cultures; (Figure S2) batch bioleaching of metals from FA using nonadapted and adapted *A. ferridurans* cultures; (Figure S3) semicontinuous bioleaching of metals from FA in a stirred tank reactor using gradually adapted *A. ferridurans* cultures; (Figure S4) PCA plot showing proteome replicate similarities of *A. ferridurans* during bioleaching of metals from MSWI residues; (Table S1) precursor range windows for diaPASEF mode; (Table S2) identified proteins in *A. ferridurans* during bioleaching of metals from MSWI residues; (Table S3) differentially expressed proteins in *A. ferridurans* during bioleaching of metals from MSWI residues; (Table S4) enriched Gene Ontology terms in *A. ferridurans* during bioleaching of metals from MSWI residues (PDF)
diaPASEF parameters (XLSX)
Gene and protein names (XLSX)
Gene and protein names (XLSX)
Gene Ontology (GO) enrichment analysis (XLSX)

## Abbreviations

ABC: ATP-binding cassette superfamily
BA: bottom ash
BSM: basal salt medium
CSTR: continuous stirred tank reactor
DIA: data-independent acquisition
diaPASEF: data-independent acquisition–parallel accumulation serial fragmentation
DEPs: differentially expressed proteins
FA: filter ash
ICP-MS: inductively coupled plasma mass spectrometry
KA: kettle ash
LC–MS/MS: liquid chromatography–tandem mass spectrometry
MFS: major facilitator superfamily
MSWI: municipal solid waste incineration
RISC: reduced inorganic sulfur compounds
RND: resistance-nodulation-cell division family
XRD: X-ray diffraction
