# Supplementary material for: Proteomic Insights into the Adaptation of Acidithiobacillus ferridurans to Municipal Solid Waste Incineration Residues for Enhanced Bioleaching Efficiency
Source: J Proteome Res. 2025 Apr 9;24(5):2243–55. doi: 10.1021/acs.jproteome.4c00527 (PMC12053936; doi:10.1021/acs.jproteome.4c00527)
Supplement: Supplementary file 1 — pr4c00527_si_001.pdf [file pr4c00527_si_001.pdf]

# Supporting Information

## Proteomic insights into the adaptation of *Acidithiobacillus ferridurans* to municipal solid waste incineration residues for enhanced bioleaching efficiency

Jiri Kucera[1]‡*, Klemens Kremser[2]‡, Pavel Bouchal[1], David Potesil[3], Tomas Vaculovic[4], Dalibor Vsiansky[5], Georg M. Guebitz[2], Martin Mandl[1]

[1] Department of Biochemistry, Faculty of Science, Masaryk University, Brno 625 00, Czech Republic; [2] University of Natural Resources and Life Sciences Vienna BOKU, Department of Agrobiotechnology, IFA-Tulln, Institute of Environmental Biotechnology, Tulln and der Donau 3430, Austria; [3] Proteomics Core Facility, Central European Institute for Technology, Masaryk University, Brno 625 00, Czech Republic; [4] Department of Chemistry, Faculty of Science, Masaryk University, Brno 625 00, Czech Republic; [5] Department of Geological Sciences, Faculty of Science, Masaryk University, Brno 611 37, Czech Republic

*Corresponding Author – jiri.kucera@sci.muni.cz (JK)

‡ These authors contributed equally.

## Contents

**Figure S1:** Batch bioleaching of metals from MSWI residues using nonadapted *A. ferridurans* cultures.
**Figure S2:** Batch bioleaching of metals from FA using nonadapted and adapted *A. ferridurans* cultures.
**Figure S3:** Semicontinuous bioleaching of metals from FA in a stirred tank reactor using gradually adapted *A. ferridurans* cultures.
**Figure S4:** PCA plot showing proteome replicate similarities of *A. ferridurans* during bioleaching of metals from MSWI residues.
**Table S1:** Precursor range windows for diaPASEF mode.
**Table S2:** Identified proteins in *A. ferridurans* during bioleaching of metals from MSWI residues.
**Table S3:** Differentially expressed proteins in *A. ferridurans* during bioleaching of metals from MSWI residues.
**Table S4:** Enriched Gene Ontology terms in *A. ferridurans* during bioleaching of metals from MSWI residues.

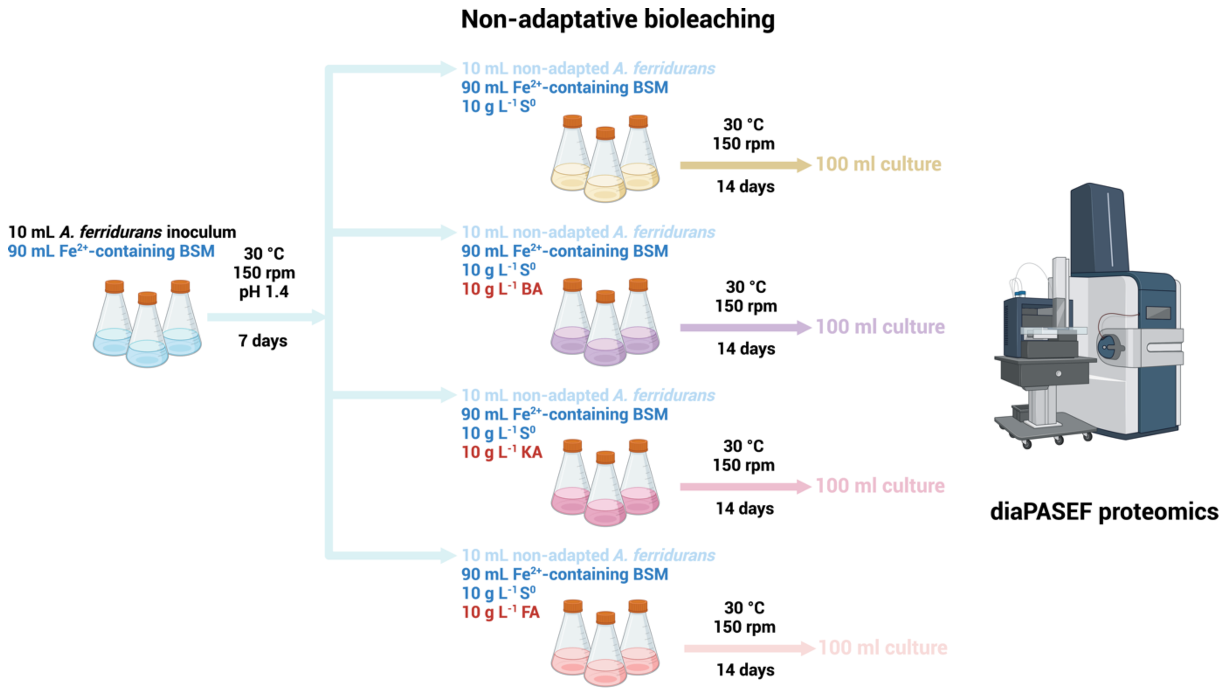


**Figure S1:** Schematic representation of the batch bioleaching of metals from various municipal solid waste incineration residues using nonadapted *A. ferridurans* cultures.

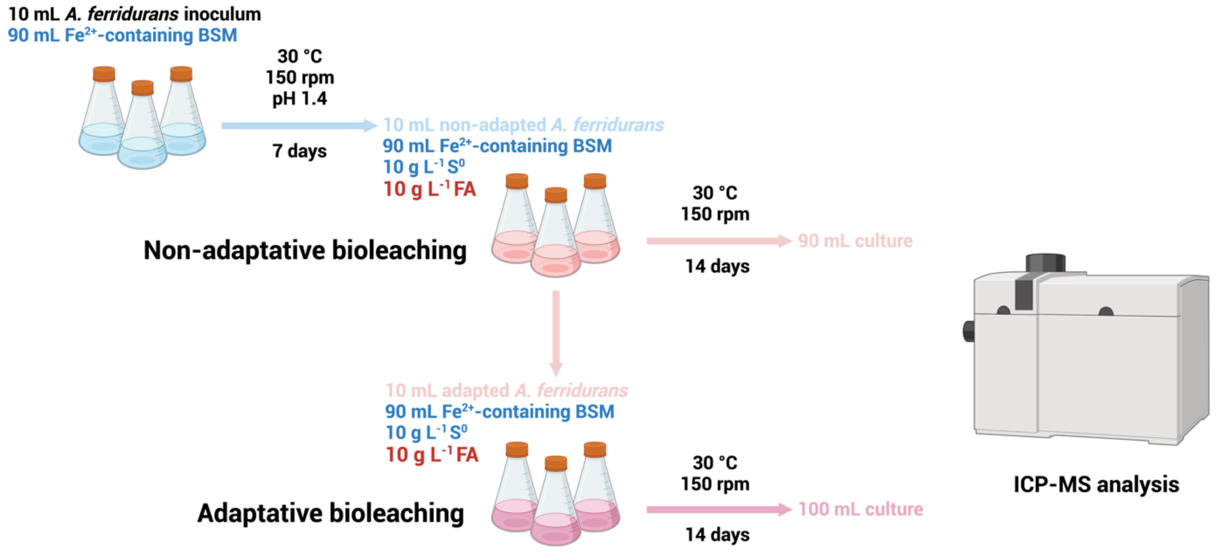


**Figure S2:** Schematic representation of the batch bioleaching of metals from filter ash using nonadapted and adapted *A. ferridurans* cultures.

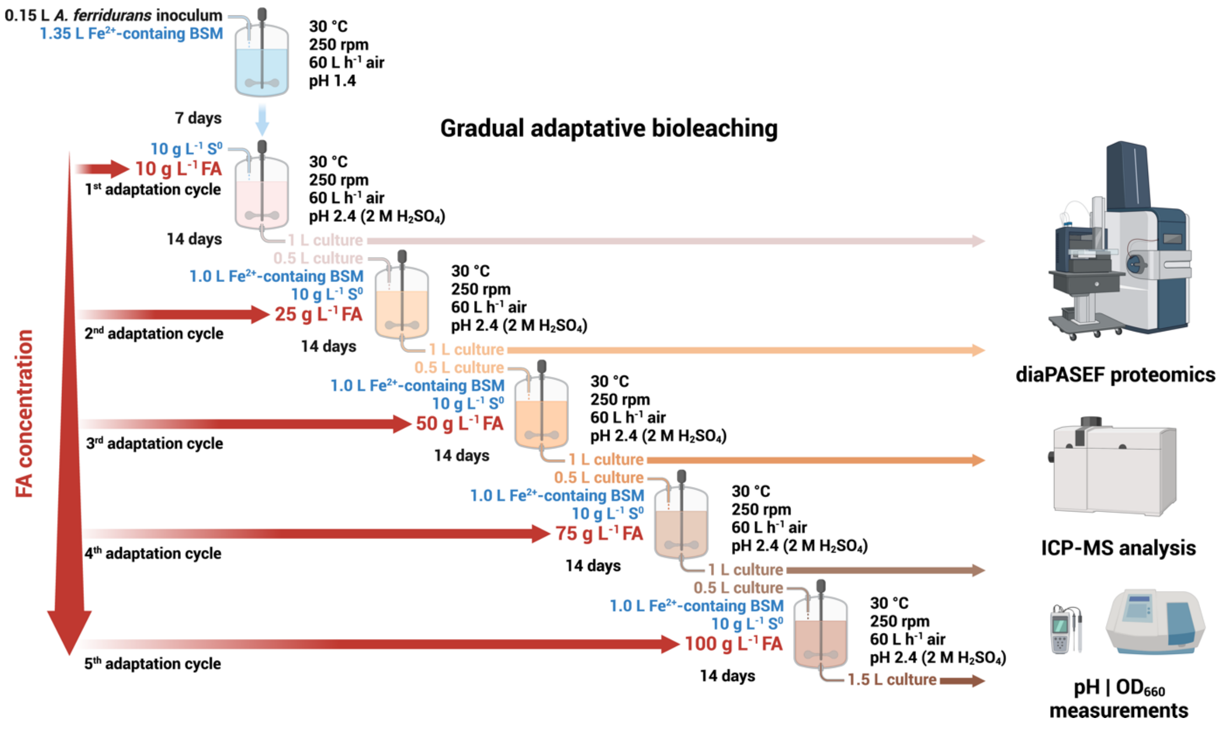


**Figure S3:** Schematic representation of the semicontinuous bioleaching of metals from filter ash in a stirred tank reactor using gradually adapted *A. ferridurans* cultures.

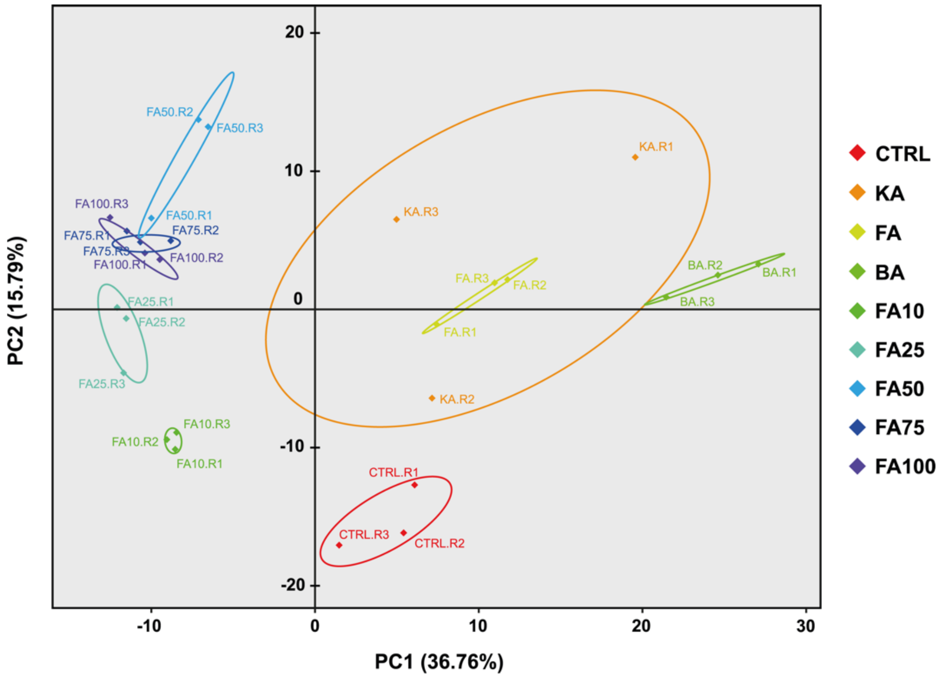


**Figure S4:** Principal component analysis plot showing similarities among proteome replicates of *A. ferridurans* during batch and semicontinuous bioleaching of municipal solid waste incineration residues.
